# Affiliative Subgroups in Preschool Classrooms: Integrating Constructs and Methods from Social Ethology and Sociometric Traditions

**DOI:** 10.1371/journal.pone.0130932

**Published:** 2015-07-02

**Authors:** António J. Santos, João R. Daniel, Carla Fernandes, Brian E. Vaughn

**Affiliations:** 1 William James Center for Research, ISPA-Instituto Universitário, Lisbon, Portugal; 2 Auburn University, Auburn, United States of America; University of Portsmouth, UNITED KINGDOM

## Abstract

Recent studies of school-age children and adolescents have used social network analyses to characterize selection and socialization aspects of peer groups. Fewer network studies have been reported for preschool classrooms and many of those have focused on structural descriptions of peer networks, and/or, on selection processes rather than on social functions of subgroup membership. In this study we started by identifying and describing different types of affiliative subgroups (HMP- high mutual proximity, LMP- low mutual proximity, and ungrouped children) in a sample of 240 Portuguese preschool children using nearest neighbor observations. Next, we used additional behavioral observations and sociometric data to show that HMP and LMP subgroups are functionally distinct: HMP subgroups appear to reflect friendship relations, whereas LMP subgroups appear to reflect common social goals, but without strong, within-subgroup dyadic ties. Finally, we examined the longitudinal implications of subgroup membership and show that children classified as HMP in consecutive years had more reciprocated friendships than did children whose subgroup classification changed from LMP or ungrouped to HMP. These results extend previous findings reported for North American peer groups.

## Introduction

After a somewhat uncertain beginning in the latter decades of the 20th century [1, 2, 3], social network analyses of school-age children and adolescents groups have proliferated rapidly in the past decade [4, 5, 6]. In part, this increase in productivity has been driven by methodological advances that untangle peer selection and influence/socialization processes within the peer group [7, 8] and show how these processes account for individual changes in psychological qualities and behavioral choices over time [9, 10, 11, 12, 13].

Although data on structural and functional aspects of school-age children and adolescents groups is increasing exponentially, studies focused on early childhood social groups remain scarce [14, 15, 16, 17, 18]. This despite dramatic increases in participation in center-based group care during early childhood (nominally 3–6 years-of-age) over the last 20 years. Moreover, most studies have focused on the utility of different methods for identifying and validating structural features of young children’s social networks, with few replications being reported. Only very recently have studies started to examine the outcomes associated with membership in specific subgroups within preschool peer group structures [19].

There are several reasons for the slow pace of research on preschool peer groups, with the most prominent being the resource demands required to obtain reliable network data. Whereas for school-age children and adolescents large groups of children may be assessed on a single occasion using some form of self-reporting [1, 6, 10], this approach is problematic with preschool children because they are typically unreliable as sources of network information [20, 21]. Consequently, the method of choice for obtaining social network data in preschool classrooms has been direct observations. These observations usually entail watching individual children many times over the course of several days or weeks and recording which of their peers are in close proximity and/or to whom they give (or receive) interactive bids [19, 21, 22]. Moreover, preschool classrooms tend to be smaller than classrooms in elementary and secondary schools, so many more classrooms must be observed to obtain similar sample sizes.

Although direct observations of young children are time consuming, the use of observational methods links this work to a rich history of ethological research on animal behavior, especially from the tradition of social ethology which puts special emphasis on social behavior and on both affiliative and dominance relationships among group members, as these structure group life across the lifespan, both within and across sex boundaries [23, 24, 25, 26, 27]. Within this literature, there has been an abiding interest in networks of affiliative social relationships [28, 29, 30] that reflect lasting social bonds. More recently, these bonds have been characterized as “friendships” between animals because they are equivalent in many respects to human friendships [31]. Findings from primate studies of social networks and friendships suggest that reciprocated social connections within the group are associated with a range of positive outcomes including better health, greater reproductive success, and support in agonistic encounters [32, 33, 34, 35]. Interestingly analogous outcomes (e.g., health, life success) have been reported in studies of social network benefits for human adults [36, 37]. The results of early social-ethological studies showing that social relationships and group structures influenced the frequency and quality of dyadic transactions motivated new studies of peer interactions and relationships of preschool age children. These studies were intended to describe the dominance and affiliative structures characteristic of preschool classrooms and to characterize both the emergence/maintenance of these structures and the constraints/affordances such structural features placed on young children’s social transactions see [38; 39] for reviews.

Strayer and associates [20, 40, 41] pioneered the application of social ethological concepts, methods, and analytic approaches to the study of preschool peer group structures. Many of the studies of primate social organization that those studies were carried out in the field where it was not always clear whether or not animals were socially engaged. To circumvent this problem, primatologists frequently used proximity (or “association”) data [42, 43] (in lieu of, or in addition to, the frequency, duration, and patterning of interactions [40] when describing social networks in primate groups. Proximity data were usually organized as association matrices to reveal which dyads were seen together at rates higher than chance, and later to identify social units (or subgroups) using cluster analytic procedures [44, 45]. Association matrices and cluster analysis proved valuable to social ethologists as they began to observe preschool age children, whose verbally elicited preferences for specific peers often did not closely match their observed proximity or interaction frequencies [20].

Strayer and Santos [17] were the first to use hierarchical cluster analyses of proximity and interaction matrices to identify subgroups (and ungrouped children) within young children’s classrooms. These subgroups reflected similarities in the patterning of partner choices and were found in all of the 15 classrooms studied. Strayer and Santos [17] argued that these subgroups could be major influences in the socialization experiences of young children, by providing the foundation for construction of a social identity or primary social reference group [38]. In a subsequent study, Santos et al. [15] distinguished two subgroup types (i.e., cliques and aggregates—according to whether children within the same subgroup were seen together at rates higher than chance). Santos et al. [21] referred to these types as “high mutual proximity” (HMP) and “low mutual proximity” (LMP) and showed that between-subgroup differences in the level of mutual investment between subgroup members (i.e., in-group preferences) were also observed for other behavioral and sociometric choice variables. The social ethology tradition continues to influence studies of peer group structures during early childhood [15, 21, 44, 45, 38]. The present study is an attempt to extend several key findings concerning the nature of subgroups within preschool classrooms [17, 21] and to further characterize the social functions associated with subgroup membership using a sample of Portuguese children.

The first goal of this study was to link the social ethological methods and measures with the considerations of child social structure in mainstream child psychology. The sociometric tradition initiated by Moreno [46] gave rise to the modern techniques of social network analysis (used by sociologists and social-ethologists), and was also foundational for research on the social relationships of children. Common sociometric tasks are used to group young children (receiving similar number of liking and disliking nominations from their peers) in different sociometric categories (e.g., popular, rejected, neglected) or to compute sociometric indices (e.g., peer acceptance) that are indicative of the relative centrality of children within their peer groups [47]. Although sociometric categories may be thought of subgroup types [48] that combine children who share a common attribute (peer acceptance and/or peer rejection), these subgroups do not imply relational ties between children (contrary to logic of the cluster analytic procedures described above). Indeed, children in a given sociometric category are not necessarily acquainted and need not be preferred social partners, even if they are acquainted. In a previous study, Santos et al. [21] found that most of the subgroups identified using cluster analyses of proximity matrices included children from different sociometric categories, indicating that the selection of social partners does not necessarily depend upon children’s social status. Although HMP and LMP subgroups both included children from different sociometric categories, children in HMP subgroups tended to have higher sociometric acceptance scores than did children in LMP subgroups, suggesting that HMP subgroups might be more socially central. Thus, the first aim of this study is to test the generality of these findings in a Portuguese preschool sample.

The second goal of the study was to extend previous findings concerning the functional distinction of HMP and LMP subgroup types. Santos et al. [21] found in-group preferences in terms of interaction frequencies, visual attention given to peers, and sociometric friendship choices, to be higher for HMP than for LMP subgroup children, supporting their characterization of LMP subgroups as having a less coherent social organization than HMP subgroups. They suggested that HMP subgroups are more likely than their LMP counterparts to be made up of friends who frequently interact with each other. To avoid a major challenge faced by [21]- namely the proliferation of chi-square tests required to demonstrate in-group preferences- we tested for HMP and LMP differences using multi-level regression models. This analytical strategy had two main advantages. First, these models allow for tests of subgroup type differences as well as effects of other relevant predictors (subgroup sociometric acceptance, subgroup sex composition) [21] in a more comprehensive way. In this study, in-group preferences are tested using a single multi-level regression model for each dependent variable. Second, it allowed us to include longitudinal data (from more than one school year) to assess changes for in-group preferences across time.

The third goal of this study concerns the social functions of subgroup membership. Because most classrooms were observed in consecutive years (same teacher, same classroom, few children joining or leaving), we could test whether membership in a particular type of subgroup had observable effects on individual level attributes that carried over from one year to the next. There is some evidence that the overall proportion of children in HMP subgroups increases with increasing age [49]. If there is a normative trend for children to form new HMP subgroups or to join an existing one, and if at the core of HMP subgroups are friendship relations [21], than children with a continuous history of membership in HMP subgroups should have more reciprocal friends than children who change subgroup type from year to year. Documenting this would support interpretations of early childhood peer experiences as fundamental influences on the social adaptation of young children.

## Method

### Participants

Data for this study were collected in nine different classrooms from two preschools serving a middle class population in the region of Lisbon, Portugal (N = 240, 120 boys, 120 girls). The children were participating in a larger longitudinal study of preschool children’s social development. All families had European backgrounds. Classroom sizes ranged from 20 to 27 children (participation rates > 70% in all classes, median: 92%), with the proportion of girls ranging between .32 and .60. Classrooms were observed one time (two classrooms), twice (four classrooms) or three times in consecutive years (three classrooms), with 88 children being observed once, 94 twice, 53 three times, and five children were observed four times (for a total of 455 cases spread across 19 classrooms). For the most part, classrooms were homogeneous for age and were categorized as 3-year-olds (i.e., children < 48 months of age at the start of the academic year, 69 girls, 73 boys), 4-year-olds (i.e., children between 48 and 60 months of age at the start of the academic year, 72 girls, 71 boys) or 5-year-olds (i.e., children between 60 and 72 months of age at the start of the academic year, 88 girls, 82 boys). In classrooms followed across consecutive years, the teacher remained the same and 83% of the children (on average) remained together from one year to the next. Written consent for children’s participation was obtained from school directors, teachers, and parents prior to data collection. The project was approved by the Portuguese Data Protection Authority (CNPD, nº 1379/08).

### Procedures

Observation and sociometric assessments were collected in tandem. For about half of the classrooms, observation data were collected before the sociometric interviews were completed, and for the remaining classrooms, sociometric interviews were conducted before the behavioral observations took place.

#### Observations

Teams of two observers independently collected focal observations for: (1) interaction (15 s duration), (2) visual attention (6 s duration), and (3) proximity (15 s duration) data in each class. Observers did not work in pairs and rarely observed a given child at the same time. Rounds of the three types of observational data were randomly interspersed. One observational “round” meant observing each child present once for one type of data (e.g., proximity). Each observer collected 100 observation rounds for each type of the three observation categories. No child present in a given day was observed twice before all other peers were observed once.

For each classroom, observations were made over a four to six week period, depending on the schedules of observers and on absences of participating children, with each observer making up to 30 focal observations of each participating child per day of observation. Observations were made at different times of day over the observation period and children were observed across the full range of classroom activities, as well as outdoor play periods. All participating children were present for a minimum of 50% of observational rounds (median number of rounds present 83%, ~160 focal observations per child per observed category). The investigators’ experience with this data collection protocol suggests that 100 observation intervals per child is the minimum number required to get a representative sample of his/her classroom proximity profile. Because most children were absent for some portion of the observation period, we set our target number of classroom observation rounds at 200.

For interaction data, observers watched each child present for a 15 s interval and at the end of the interval recorded identifying information for every child with whom the target interacted. Codes for the initiator and target of the interaction and affective valence (positive, neutral and negative) of the interaction were recorded. The valence was coded as positive if one or both children showed positive affect (e.g., gesture, smile, laugh, or vocalization indicative of positive affect) in the context of the social exchange, unless this expression was accompanied by expressions of negative affect from the interactive partner. The valence was coded as negative if one or both children expressed negative affect (e.g., distress, anger, fear, sadness), in a facial, gestural, or vocal mode, unless those expressions were made in the context of fantasy play. Any exchange not coded as positive or negative was coded as neutral (e.g., greetings or conversations during a meal or in the context of a task that did not include an affective expression, nonverbal exchanges that included physical contact and a response to contact). For some observation intervals an interaction was ongoing when the observer reached a target’s identifier on the classroom roster. In such cases, observers were instructed to identify the first initiated exchange occurring during the observation interval and to code the child who initiated the exchange as the initiating child for the interval.

To obtain visual attention data, observers watched a target child for a 6 s interval and recorded the identity codes for all children receiving a unit of visual regard from the observation target (where a visual regard “unit” was defined as the orientation of head and eyes toward the peer recipient). Each recipient of visual regard was credited with a single unit per 6 s observation interval.

Social proximity information was collected as the child’s “nearest neighbor” for each interval. Each child present in the classroom was observed for a 15 s interval, at the end of which the child’s nearest peer neighbor was identified. A child who was within arm’s reach (if both children were to reach out, roughly 3–4 feet) and engaged in the same or a similar activity as the observation target was considered his/her nearest neighbor. If two or more children were equally nearby to the focal child (as could happen when children were engaged in table activities or in group time) the child to the target’s immediate right was considered the nearest neighbor. For instances in which the target was interacting verbally or physically with a child at the end of the observation interval, that interaction partner was coded as the nearest neighbor, even though another child might have been physically closer.

Observers were trained to 80%+ agreement prior to data collection in live observations. For interaction and visual regard observations, observer agreement was estimated as the intra-class correlation (ICC) of individual rate scores across observers. For nearest neighbor data, the ICC was estimated from the dyadic co-occurrence profiles. Median ICC estimates for each pair of observers were .52 for positive interactions, .74 for neutral interactions, and 76 for visual attention. For the nearest neighbor data, the ICC was .85. Rates of negative interactions were too low to be used in this study (social attention: M = .51, SD = .22; positive interactions: M = .10, SD = .08; neutral interactions: M = .34, SD = .15; negative interactions: M = .03, SD = .03).

#### Sociometric measures

Participants completed three sociometric tasks, using photos of classmates as the choice stimuli: (a) positive and negative nominations; (b) paired comparisons; and (c) rating scale. For each task, children were queried about all classmates (both boys and girls). The assessments took place outside of the classroom in a quiet area. Typically, the nominations task was administered first, followed by the rating-scale task. The paired comparison measure was always administered last. The sociometric interviews were completed in 30–45 min (typically two or three 15 min sessions). If a child’s attention wandered, the interviewer stopped the task and continued it at another time.

For the nominations task, children were presented with the array of photographs of all classmates and asked to name each one. After successfully naming all classmates, the child was asked to identify a peer with whom she or he especially liked to play. The request was repeated two more times and then the child was asked to identify a peer with whom she or he did not especially like to play (again repeated twice). For the rating-scale task, the child was presented with photographs of classmates in a random order and asked to sort participating classmates’ photos into one of three containers: children with whom the child liked to play a lot, sort of liked to play, or did not like to play (scored 3, 2, 1 respectively). The child was also asked to verbalize his or her choices. For the paired comparisons task all possible pairs (total number of comparisons in a given classroom = N(N-1) / 2) were shown to the child being interviewed. The child was asked which of these two children do you especially like to play with?, for each pair. Pairs were ordered such that all children in a given group were seen once before any child was seen twice and each child’s photograph appeared an equal number of times on the left and right hand sections of the stimulus cards.

### Data Analysis

#### Affiliative subgroups

For each classroom, children were assigned rows in a symmetric co-occurrence matrix (oij = oji) and the numbers of times two children were observed to be nearest neighbors were matrix columns. Pearson correlations were used as indices of similarity between pairs of proximity profiles (i.e., lines of the co-occurrence matrix) for each dyad. These similarity values were then submitted to hierarchical cluster analysis using a complete linkage algorithm. The complete-linkage clustering, also designated as the furthest neighbor sorting, was chosen because this algorithm makes it difficult to assign new members to an existing subgroup at each consecutive step of the clustering process. This results in greater profile similarity among cluster co-members. A within-cluster correlation coefficient at the *p* < .05 level of significance was used to identify subgroups (i.e., children whose dyadic association patterns across the full peer group were similar) vs. ungrouped cases (i.e., children whose proximity profile was not significantly correlated with the profile of any of his/her peers). Next, we split the subgroups according to the level of mutual proximity among co-members to identify high vs. low mutual proximity subgroups. A subgroup was considered to show high mutual proximity (HMP) if all subgroup members associated with each other above chance levels (> χ2(1) with α = .001). For example, in a three child subgroup, all three χ^2^(1) tests needed to be significant (observed = ∑_*j*_ *o*_*ij*_ for every subgroup member j; expectedi = $(n-1)\times \bar{o_{ij}}$, where n equals subgroup size). If *p* > .001for any of these tests, the subgroup was categorized as low in mutual proximity (LMP).

#### Sociometric status categories

Following [50] we used positive and negative sociometric nominations to derive popular, neglected, and rejected (the remaining children were classed as average) status categories. Subgroups were considered “pure” (e.g., pure-popular, pure-rejected) if all members of the subgroup had the same sociometric status, mixed-P if at least one child in the subgroup was classified as popular and mixed not-P if no child in the subgroup was classified as popular.

#### Stratification of subgroups by social acceptance

A peer acceptance score of each child was calculated by the ratio between the total number of choices received from classmates in the paired comparisons task and the number of classmates completing the task. An acceptance score was then calculated for every subgroup by averaging subgroup members’ peer acceptance scores. These subgroup scores were converted to percentile ranks (PR) within classroom and subgroups were then categorized as having low (PR < 50), or high acceptance.

#### Friendship choices

Following [51], to be considered as a friend a peer had to appear among the upper quintile on either the nominations or the paired comparisons sociometric tasks and had to receive a rating of 3 (“like to play with a lot”) on the rating scale task. We used this sequential criteria rule to assure that children were being consistent in naming preferred classmates because preschool children often use different criteria for identifying “friends” than older children and adolescents [51]. If a given child was also chosen as a friend (as defined above) by a peer he/she had chosen, the dyad was categorized as a “reciprocated friendship dyad.”

#### In-group preference

For each member of a multi-child subgroup, we computed the proportion of social attention, positive and neutral interactions, and sociometric friendship choices given to group members to index in-group preferences. Multilevel regression models were used to compare in-group preference across time (t = 0 for 3-year-olds, t = 1 for 4-year-olds and t = 2 for 5-year-olds), subgroup type (HMP, LMP), subgroup sex composition (same-sex, mixed-sex), subgroup acceptance (high, low), controlling for subgroup size. Because the dependent variables are proportions we used generalized linear mixed models, with a logit link and a binomial error distribution [52]. All models were two level-models created with the repeated measures at the lowest level and the individual children at the highest level. Despite the small numbers of observations for each children multilevel models are very effective in detecting fixed effects of model predictors [53] and do not require the same number of measurements for all individuals in order to obtain efficient estimates [52].

#### Longitudinal implications of subgroup membership

Our analyses detected a normative movement of children into HMP subgroups with increasing age (see Results below). Consequently, it was possible to compare children who continuously occupy HMP subgroups in consecutive years and their peers who became HMP subgroup members after having been ungrouped or in LMP subgroups in the previous year. For this analysis, we used the total number of reciprocated friendships as the dependent variable and controlled both the number of reciprocated friendships in the previous years and changes in classroom size (which might influence the possible number of reciprocated friendships possible in a given classroom). This analysis was restricted to HMP subgroups because too few children were classified as belonging to LMP subgroups or being ungrouped in consecutive years.

## Results

### Descriptive Analyses

#### Affiliative subgroups

The cluster analyses identified a total of 146 multi-child subgroups and 37 children ungrouped. 110 (75.3%) subgroups were subsequently classified as high mutual proximity (HMP), and 36 (24.7%) were classified as low mutual proximity (LMP). HMP subgroups were observed in every classroom, LMP subgroups were found in 14 of 19 classrooms, and ungrouped children were present in 16 classrooms. No classroom contained subgroups of only one type. Approximately 74% (108/146) of multi-child subgroups only included same-sex children. Boys and girls were equally represented in each subgroup type, χ2 (2, *N* = 455) = 5.10, *p* = .08. *V* = .11 (Table 1). Approximately 43% (total = 63/146, HMP = 45/110, 41%; LMP = 18/36, 50%) of all multi-child subgroups were dyads (mixed-sex = 15/63, 24%; boys = 20/63, 32%; girls = 28/63, 44%) and these accounted for nearly 30% (126/418) of grouped children. Ten HMP subgroups had more than four children and one HMP subgroup included six children, however, an ANOVA on subgroup size, using age, subgroup type, subgroup sex composition, and subgroup sociometric stratification level as predictors, did not reveal any significant main effects or interactions (subgroup size: *M* = 2.86, *SD* = .94).

**Table 1:** Number of Boys and Girls Present in each Affiliative Subgroup.

Subgroup type	Girls (*n* = 229)	Boys (*n* = 226)	Total (*N* = 455)
HMP	153	172	325
LMP	53	40	93
Ungrouped	23	14	37

The proportion of children belonging to each subgroup type varied as a function of age, χ2 (4, *N* = 455) = 11.58, *p* = .02, *V* = .11 (Table 2). HMP subgroups were more common among 5-year-olds. Mixed sex subgroups were slightly more common in classrooms of 3-year-olds (38%) than in classrooms of either 4- (27%) or 5-year-olds (15%), χ^2^(2, *N* = 146) = 8.12, *p* = .09. *V* = .17. Subgroup sex composition (i.e., all female, all male, mixed), χ^2^ (2, *N* = 146) 1.34, *p* = .53, *V* = .10, was not significantly associated with subgroup type.

**Table 2:** Distribution of Children Within Affiliative Subgroups by Age Level.

	Subgroup type		
Age group	HMP (*n* = 325)	LMP (*n* = 93)	Ungrouped (*n* = 37)
3 (*n* = 142)	91	39	12
4 (*n* = 143)	98	31	14
5 (*n* = 170)	136	23	11

#### Sociometric status and subgroup acceptance

At the level of individual children, average and popular sociometric status were under-represented in ungrouped children, whereas rejected and neglected status children were over-represented in the ungrouped category χ^2^ (6, *N* = 410) = 19.65, *p* < .01, *V* = .16 (Table 3). The cross-tabulation presented in Table 3 indicated that subgroup type (HMP vs. LMP) and sociometric subgroup status composition (i.e., all subgroup members from a single sociometric status group vs. mixed status groups with at least one popular case vs. mixed status groups without any popular members) were independent of each other, χ^2^ (3, *N* = 112) = 3.76, *p* = .28, *V* = .18. Approximately 75% of the subgroups included children from different sociometric status categories. No HMP or LMP subgroup was homogeneous for popular or rejected status children. Ungrouped children were not included in this chi-squared analysis because it was structurally impossible for them to appear in a “mixed-“categories. Twenty-six of the 28 pure status subgroups were made up of average status children and two included only neglected children (Table 4).

**Table 3:** Subgroup Type and Children Sociometric Status.

	Sociometric status			
Subgroup type	Popular	Average	Neglected	Rejected
HMP	37	194	34	33
LMP	13	45	14	11
Ungrouped	1	12	6	10

**Table 4:** Subgroup Type and Subgroup Sociometric Status Classifications.

	Subgroup sociometric status					
Subgroup type	Mixed P	Mixed non-P	Popular	Average	Neglected	Rejected
HMP	28	32	0	23	1	0
LMP	11	13	0	3	1	0

*Note*. Subgroups were classified has mixed-P if at least one child in the subgroup was popular and mixed not-P if no child in the subgroup was popular. Remaining categories refer to subgroups including children with the same sociometric status.

The cross-tabulation of subgroup type by sociometric acceptance level (high acceptance vs. low acceptance) was not significant, χ^2^(2, *N* = 174,) = 2.93, *p* = .23, *V* = .13. Together this set of analyses suggest that there is no direct correspondence between the implicative categories of sociometric status (i.e., popular, rejected, neglected) or the acceptance level of subgroups and subgroup type, *per se*, for children in multi-child subgroups.

#### In-Group Preference

**Fig 1:**
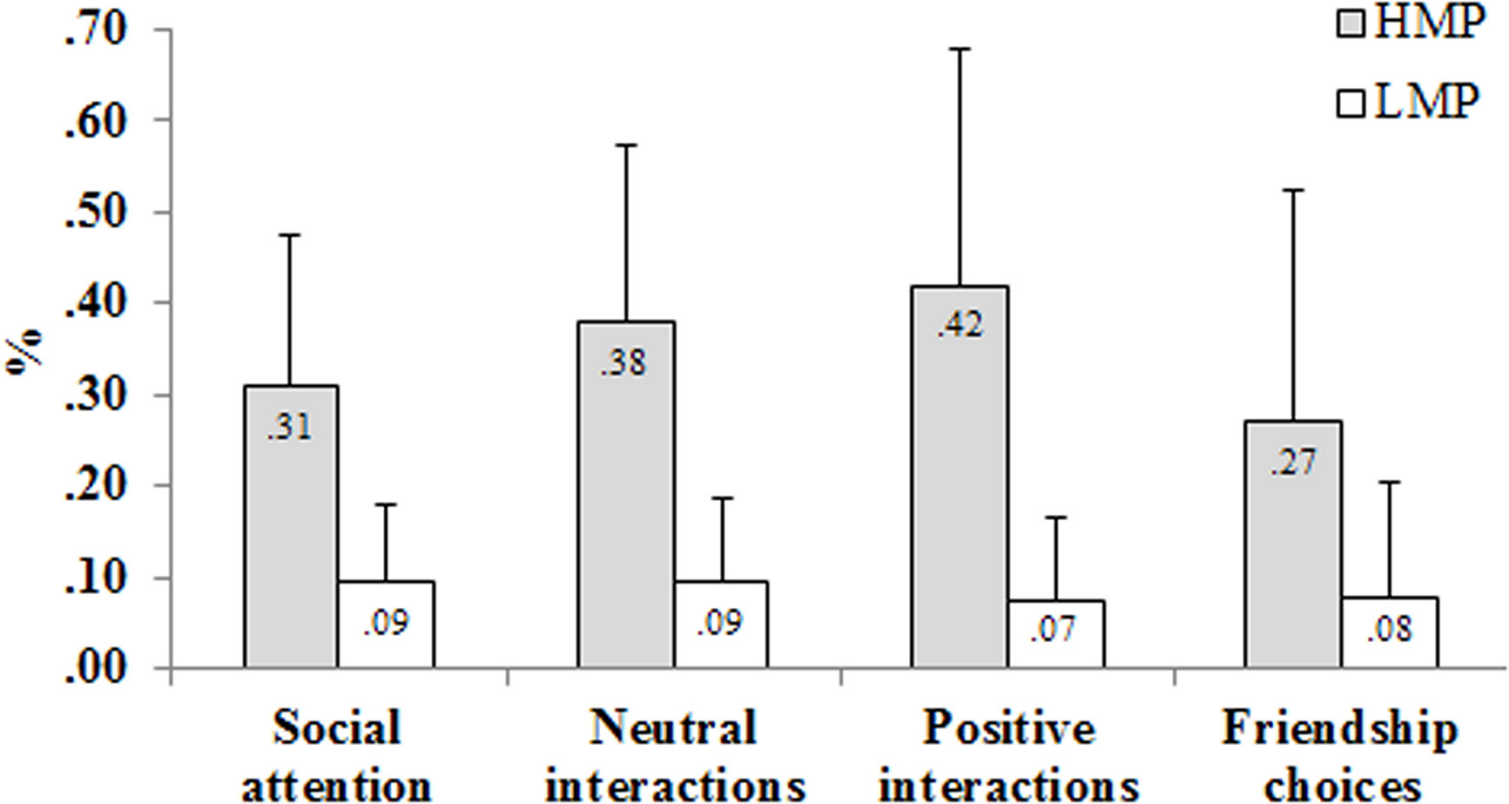
Proportion (*M + SD*) of social attention, neutral interactions, positive interactions and friendship sociometric choices directed to subgroup members according to subgroup type (HMP–high mutual proximity, LMP- low mutual proximity).

Table 5 presents regression estimates from the multilevel models of in-group preference (controlling for subgroup size). Subgroup type (HMP vs. LMP) had a significant effect on all dependent variables studied. Children in HMP subgroups were more likely to direct social attention (odds-ratio = 3.39), neutral (odds-ratio = 5.00) and positive interactions (odds-ration = 8.08), and friendship choices (odds-ratio = 2.59) to their co-members than did children in LMP subgroups (Fig 1). Subgroup sex composition was significantly related to the distribution of social attention, and neutral and positive interactions- children in same-sex subgroups showed more in-group preference than children in mixed-sex subgroups (odds-ratios = 1.19, 1.72, 1.43 respectively)- but not for friendship sociometric choices (odds-ratio = 1.12). Subgroup sociometric acceptance level categorized as low *vs*. high acceptance had a significant effect on friendship choices. In-group preferences for friendship were significantly higher in the high acceptance subgroups (odds-ratio = 1.54). The in-group preference scores for social attention, neutral interactions, and positive interactions were in the same direction but did not reach significance (odds-ratios = 1.08, 1.12, 1.21 respectively).

**Table 5:** In-Group Preferences Multilevel Models (*β ± SE*).

	Social attention (*n* = 414)	Neutral interactions (*n* = 312)	Positive interactions (*n* = 308)	Friendship choices (*n* = 354)
Intercept only model				
*Y*_*ti*_ = logistic (*β*_*0i*_ + *e*_*ti*_); *β*_*0i*_ = *β*_*00*_ + *u*_*0i*_				
Intercept (*β*_*00*_)	-1.24 (.06)**	-.89 (.07) **	-.73 (.08) **	-1.24 (.07)**
*σ* ^*2*^_*μ*_	.81	.89	1.17	.15
*σ* ^*2*^_*e*_	1.00	1.00	1.00	1.00
Deviance	2571.65	1725.17	1360.91	1185.46
Full model				
*Y*_*ti*_ = logistic (*β*_*0i*_ + *β*_*10*_Time_*ti*_ + *β*_*20*_LMP_*ti*_ + *β*_*30*_Mixed_*ti*_ + *β*_*40*_High_*ti*_ + *β*_*50*_Size_*ti*_ + *e*_*ti*_); *β*_*0i*_ = *β*_*00*_ + *u*_*0i*_				
Intercept (*β*_*00*_)	-2.24 (.09)**	-1.66 (.13) **	-1.58 (.20)**	-3.09 (.26)**
Time (*β*_*10*_)	-.16 (.02) **	-.25 (.04) **	-.36 (.07) **	.06 (.08)
SG type: LMP (*β*_*20*_)	-1.22 (.06)**	-1.61 (.10) **	-2.09 (.19)**	-.95 (.22) **
SG sex: Mixed (*β*_*30*_)	-.17 (.05) **	-.54 (.08) **	-.36 (.13) **	-.11 (.15)
SG acceptance: High (*β*_*40*_)	.08 (.05)	.11 (.08)	.19 (.12)	.43 (14) **
SG Size (*β*_*50*_)	.45 (.02) **	.45 (.03) **	.49 (.05) **	.54 (.07) **
*σ* ^*2*^_*μ*_	.40	.45	.77	.00
*σ* ^*2*^_*e*_	1.00	1.00	1.00	1.00
Deviance	1431.51	1008.41	1084.30	1148.88

*Note*. HMP, same-sex and low acceptance subgroup children were used as the reference categories for subgroup (SG) type, subgroup sex and subgroup acceptance variables respectively. Time was coded as *t* = 0 for 3-year-olds, *t* = 1 for 4-year-olds and *t* = 2 for 5-year-olds. To make the interpretation of the regression coefficients easier, these can be transformed to odds-ratios: odds-ratio = exp(*β*).

* *p* < .05

** *p* < .01

Time was associated with decreasing in-group preferences for the observational measures. The direction of these differences was not anticipated, and so additional analyses tested the main effect of time on rates of visual attention given to all peers as well as positive and neutral interactions initiated to all peers. In these analyses, rates of social attention decreased significantly over successive years of observation, β = -14.57, SE = 1.79, p < .001, whereas rates of initiated neutral, β = 8.13, SE = 1.09, p < .001 and positive interactions, β = 3.45, SE = .61, p < .001, increased. Thus, while the negative change with regard to visual attention to group co-members is consistent with age trends for the individual classrooms, the changes for initiated interactions remain counter-intuitive.

#### Longitudinal Implications of Subgroup Membership

Children in HMP subgroups in consecutive years had more reciprocated friendships (M = 1.78, *SD* = 1.31) than did their HMP peers who had moved from either LMP subgroups or ungrouped status in the previous year (*M* = 1.17, *SD* = 1.16), *F*(1, 146) = 7.29, *p* < .01. The change in classroom size covariate and reciprocated friendships score of the previous were also significant in this analysis (*p* < .01). This result suggests that continuous occupation of HMP subgroups is associated with increases in close friendships over time.

## Discussion

This study had three main goals. The first was to determine whether the different types of preschool affiliative subgroups that have been described for North American samples would also be found in this Portuguese preschool sample. The second was to test for in-group preferences in terms of social attention, initiated interactions, and sociometric choices that might distinguish the two subgroup types (i.e., LMP vs. HMP; ungrouped cases could not show an in-group preference) and suggest functional differences between the subgroup types. The third goal was to examine the longitudinal implications of subgroup membership with reference to children’s reciprocated friendships.

Concerning the first goal, our results replicated previous findings reported for North American preschool classrooms [20] in a Portuguese preschool sample. Because Portuguese samples are underrepresented in research with young children from any tradition in social development, our study contributes to the characterization of this population and suggests that the descriptions of group structures in young children are more similar than different across cultures.

Globally, our results indicate that hierarchical cluster analyses of proximity matrices pioneered by [17] and their associates are valid procedures for identifying subgroups in preschool classrooms. Most children were assigned to a subgroup by the clustering algorithm, although 37 cases (~8% of the total) were not included in any subgroup using our criterion of a significant correlation (i.e., *p* < .05) between children’s proximity profiles across all classmates. Subgroups were further distinguished in two different types- HMP and LMP- based on the degree of association among subgroup members. All classrooms included HMP subgroups in combination with LMP subgroups and/or ungrouped children. Both sexes were equally represented in each of the subgroup types and the majority of subgroups were sex segregated. HMP subgroups were more frequent in classrooms of older children. Mixed-sex subgroups were more frequent in classrooms of younger children, supporting findings of increasing sex segregation across the early childhood years [54]. Together, these results are consistent with social-ethological assumptions that both social transactions and the child’s developmental status would be associated with the forms that social structures take during early childhood [17].

Dyads were frequent subgroups in this sample. We are aware of the ongoing debate among social network researchers concerning the status of dyads. Some [55] argue that dyads should not be considered as groups because processes often used to define groups (e.g., transitivity of ties) cannot be observed in dyads and/or that dyads frequently exhibit properties (e.g., close interpersonal relationships) that are not defining features of groups [3]. Others [56] argue equally persuasively that dyads have more in common with larger groups than differences and should be considered as legitimate groups. In [57]’s model of social structures, dyadic relationships occupy an intermediate level between interactions and social structures, but it seems clear from his arguments that overall group structures reflect relationships among dyads. This suggests that Hinde’s model includes dyads as legitimate subgroups within the larger social structure. We view dyads as the core(s) of subgroups in preschool classrooms and they are the most common form for the youngest children, suggesting that they are the first subgroup forms that can be sustained by very young children.

Overall, these findings support the use of hierarchical clustering methods to identify affiliative subgroups within classrooms. Without an algorithm that assigns individuals to a unique subgroup (i.e., non-overlapping subgroup boundaries) it would not have been possible to evaluate social preferences and interactions within and between subgroups.

Concerning the second goal, we extend previous findings of Santos, Strayer and their associates [17, 21] using a new analytical framework (multi-level regression models) that represents an advance over previous attempts at describing social preferences in groups of young children. Multi-level regression analyses showed that HMP membership was associated with higher in-group social preference using both behavioral rate scores and sociometric choices. This constitutes evidence that the socio-structural analyses using proximity data are sensitive to differences between subgroup types, and that these differences have implications for understanding the connections between affiliative networks and positive social communication that are assumed in social ethological theory [17]. To the extent that membership in a subgroup provides the aliment for construction of a social identity or primary social reference group [38], it is important to have a rationale for demarcating subgroup boundaries. Non-agglomerative algorithms that allow membership in multiple subgroups would have been less useful for this purpose. Again, this supports our use of hierarchical clustering techniques for identification of subgroups within the larger classroom groups.

Regarding children in LMP subgroups, our results show patterns of in-group preferences, which conform to expectations regarding their similar but less selective association. Finding different patterns of in-group attraction across subgroup types supports Santos et al.’s [21] argument that HMP and LMP subgroups are functionally distinct, with HMP subgroup members more likely to be friends than LMP subgroup members. This suggests differing degrees of in-group connectedness across subgroup types, which could indicate that LMP subgroups are likely to be less stable over time, with members leaving to join HMP groups. Alternatively, it may be that LMP subgroups are precursors to or even the early stages of HMP groups. We are currently collecting more data to test which of these hypotheses yields a more accurate description of the temporal dynamics of subgroups.

Stratification of subgroups according to their levels of peer acceptance revealed that the salience of co-members as targets of sociometric choices was stronger for members of high social acceptance subgroups. For members of these subgroups a dual process of propinquity/familiarity and attraction to high social acceptance may potentiate in-group sociometric attraction. Subgroup acceptance did not have a significant positive influence on in-group preferences for the social attention or interaction measures. These results, together with fact that the majority of subgroups included children with different sociometric status classifications show that group structures do not simply reflect peer likability at the group level. That is, neither “popular” nor “rejected” children in a classroom necessarily preferred each other as associates. Preschool children may not be as aware of group-level assessments as older children may be [58] and it could be that sociometric birds of a feather do flock together at later ages.

From a social developmental point of view, an important question concerns whether patterns of association depend upon conscious categorization and social comparison with members of the larger group. This question seems pertinent given the degree of sex segregation in the subgroups and the fact that, for observed variables, mixed subgroups had significantly lower in-group preference scores than did same-sex subgroups. That preschool children tend to play with same-sex peers has been documented in the developmental literature since the 1930's [59]. [60] reported that 70% of all social initiations were directed to same-sex peers and that children returned to same-sex play more rapidly than to mixed-sex play. However, partner’s behavioral style was also a significant predictor of initiation preference and for some children was more influential than sex. They reported that positive affect was nearly three times more likely with same-sex than with other-sex partners and, for girls, negative affect was approximately four times more likely when interacting with a boy. Findings reported in [60] suggest that, while demographic homophilies (e.g., same sex) may affect initial selection of subgroup members, it is the affect expressed within the subgroup that determines subgroup cohesiveness (i.e., HMP vs. LMP). The exchange and the balance of positive vs. negative affects may also contribute to subgroup changes over time (e.g., losing or gaining members). These examples illustrate how subgroups might serve as micro-socialization contexts affording opportunities for behavior exploration and appropriation by group co-members but these subgroups may also limit or otherwise constrain behavior, attitudes, and norm commitment within the subgroup.

We did not anticipate finding stronger in-group preferences among younger children for the behavioral measures and these results may be due to the less-well developed social, cognitive, and communicative abilities of younger children [59]. Younger children may tend to monitor their play partners’ availability using direct visual tracking rather than through conversation to a greater extent than do older children. Moreover, younger children may tend to allocate social initiations to a smaller proportion of the available peers than do older children. Given our finding that overall rates of visual attention to peers declined for children observed over consecutive years, either of these, admittedly speculative, reasons could lead to greater in-group visual attention preference among younger children. At the same time, interaction rates actually increased for children seen across consecutive years and the falling rate of within-subgroup preferences cannot be answered from our current dataset. New research will be needed to test these speculations.

Concerning the third goal, individual histories of subgroup membership across consecutive years did predict reciprocated friendships. Children classified as HMP in consecutive years had more reciprocated friendships than children whose subgroup classification changed from LMP or ungrouped to HMP. Because neither in-group preferences for friendship choices nor subgroup sizes increased over time, this may indicate that children participating in HMP subgroups in consecutive years become more visible and desirable as partners to children outside their subgroup, at least when class rosters do not get shuffled in consecutive years of preschool (as was the case in this sample). This speculation should be tested in future research on the impacts of subgroup membership during early childhood.

Such research might also assess the quality of reciprocated friendships for children with histories of HMP membership (compared to the reciprocated friendships of children who move from LMP or ungrouped status from one year to the next). The literature on peer influences suggests that children linked by stronger social ties tend, over time, to converge with respect to their modes of social and academic adaptation [61]. We might expect to find this convergence more commonly in HMP than LMP subgroups and it would be of considerable interest to know just how such convergence might be achieved. For example, if a child with a history of LMP membership was integrated into an HMP subgroup in a subsequent academic year, would that child be more likely to move toward peers who had continuous HMP status, or would both the former LMP and continuous HMP children both change, but in different directions so as to converge on an intermediate level of adaptation? Such a study would go beyond the structural and functional descriptions of subgroups and consider the behavioral and emotional dynamics of subgroup members.

Our results highlight an important issue relating to current debates about social dominance in children. Recent studies of dominance shifted from relational definitions to trait definitions reflecting access to and control over resources within the group [62]. Our findings showing a normative trend toward HMP groups suggest the possibility that the most valuable resources in preschool peer groups are other children and that being a member of a subgroup, especially the HMP type linking two or more friends, is likely a goal for preschool children. Our findings show that membership in an HMP subgroup benefits individual child members socially by enlarging the number of friends and potential friends in the classroom. In this sense, affiliative subgroups constitute resources and membership in more influential affiliative subgroups could also be considered an index of social dominance. Members of these subgroups may, for example, set the activity preferences in the classroom or enforce classroom norms (even though many of these norms are likely set by the adults also in the classrooms).

To conclude, we were able to replicate several important results regarding the form and function of subgroups in preschool children’s classrooms that were reported previously for North American samples [15, 17, 21], in a longitudinal sample of Portuguese preschool classrooms. Our findings that in-group preferences distinguish subgroup types extend the generality of conclusions concerning the nature and implications of peer experiences to another sociocultural context. Examination of the different subgroup types over consecutive years showed not only that there was a progression toward becoming a member of an HMP subgroup, but also that being a member of HMP subgroups in consecutive years was associated with increasing numbers of reciprocated friendships. This last result suggests that subgroup forms can influence the trajectory of a child’s success in stable social contexts during early childhood. Documenting such effects is important because they suggest that social settings beyond those present in the nuclear family can contribute in important ways to children’s adaptive functioning during early childhood.
